# Identification of QTLs associated with oil content and mapping *FAD2* genes and their relative contribution to oil quality in peanut (*Arachis hypogaea* L.)

**DOI:** 10.1186/s12863-014-0133-4

**Published:** 2014-12-10

**Authors:** Manish K Pandey, Ming Li Wang, Lixian Qiao, Suping Feng, Pawan Khera, Hui Wang, Brandon Tonnis, Noelle A Barkley, Jianping Wang, C Corley Holbrook, Albert K Culbreath, Rajeev K Varshney, Baozhu Guo

**Affiliations:** US Department of Agriculture-Agricultural Research Service, Crop Protection and Management Research Unit, Tifton, GA USA; International Crops Research Institute for the Semi-Arid Tropics (ICRISAT), Hyderabad, India; Department of Plant Pathology, University of Georgia, Tifton, GA USA; US Department of Agriculture-Agricultural Research Service, Plant Genetic Resources Conservation Unit, Griffin, GA USA; College of Life Science, Qingdao Agricultural University, Qingdao, China; College of Bioscience and Biotechnology, Qiongzhou University, Sanya, China; Peanut Research Institute, Shandong Academy of Agricultural Sciences, Qingdao, China; Department of Agronomy, University of Florida, Gainesville, FL USA; US Department of Agriculture-Agricultural Research Service, Crop Genetics and Breeding Research Unit, Tifton, GA USA

**Keywords:** Peanut, Genetic map, QTL analysis, Oil content, Oleic acid, Linoleic acid, O/L ratio, *FAD2* genes

## Abstract

**Background:**

Peanut is one of the major source for human consumption worldwide and its seed contain approximately 50% oil. Improvement of oil content and quality traits (high oleic and low linoleic acid) in peanut could be accelerated by exploiting linked markers through molecular breeding. The objective of this study was to identify QTLs associated with oil content, and estimate relative contribution of *FAD2* genes (*ahFAD2A* and *ahFAD2B*) to oil quality traits in two recombinant inbred line (RIL) populations.

**Results:**

Improved genetic linkage maps were developed for S-population (SunOleic 97R × NC94022) with 206 (1780.6 cM) and T-population (Tifrunner × GT-C20) with 378 (2487.4 cM) marker loci. A total of 6 and 9 QTLs controlling oil content were identified in the S- and T-population, respectively. The contribution of each QTL towards oil content variation ranged from 3.07 to 10.23% in the S-population and from 3.93 to 14.07% in the T-population. The mapping positions for *ahFAD2A* (A sub-genome) and *ahFAD2B* (B sub-genome) genes were assigned on a09 and b09 linkage groups. The *ahFAD2B* gene (26.54%, 25.59% and 41.02% PVE) had higher phenotypic effect on oleic acid (C18:1), linoleic acid (C18:2), and oleic/linoleic acid ratio (O/L ratio) than *ahFAD2A* gene (8.08%, 6.86% and 3.78% PVE). The *FAD2* genes had no effect on oil content. This study identified a total of 78 main-effect QTLs (M-QTLs) with up to 42.33% phenotypic variation (PVE) and 10 epistatic QTLs (E-QTLs) up to 3.31% PVE for oil content and quality traits.

**Conclusions:**

A total of 78 main-effect QTLs (M-QTLs) and 10 E-QTLs have been detected for oil content and oil quality traits. One major QTL (more than 10% PVE) was identified in both the populations for oil content with source alleles from NC94022 and GT-C20 parental genotypes. *FAD2* genes showed high effect for oleic acid (C18:1), linoleic acid (C18:2), and O/L ratio while no effect on total oil content. The information on phenotypic effect of *FAD2* genes for oleic acid, linoleic acid and O/L ratio, and oil content will be applied in breeding selection.

**Electronic supplementary material:**

The online version of this article (doi:10.1186/s12863-014-0133-4) contains supplementary material, which is available to authorized users.

## Background

Peanut (*Arachis hypogaea* L.) is mostly grown in semi-arid tropic (SAT) regions in over 100 countries of Asia, Africa and Americas. In 2012, the global production was 41.18 m tons from an area of 24.70 m ha [1]. It is one of the main oil crops of the world averaging about 50% oil content and it could be as low as less than 40% [2]. In countries such as China and India, peanuts are primarily crushed for oil, and thus increasing oil content is the breeding priority. In the United States, peanuts are primarily used as edible products (such as peanut butter, roasted and salted peanuts, confectionaries, or in-shell peanuts), and lowering the oil content is a breeding objective. High O/L ratio (ratio of oleic and linoleic acid) is the most desired oil quality trait as it provide increased shelf life and health benefits to manufacturers and consumers, respectively. Fatty acid desaturase (FAD2) catalyzes the conversion of oleic acid to linoleic acid by adding a double bond to oleic acid [3]. This enzyme is encoded by two homeologous genes, *ahFAD2A* and *ahFAD2B*, located on the A and B sub-genomes, respectively [4-6]. Both the *FAD2* genes have 99% sequence homology and inactivation of both copies of the enzymes results in high O/L ratio in mutants. The mutant *ahFAD2A* gene had substitution (G:C to A:T) and *ahFAD2B* gene had insertion (A:T) of one base pair. These mutations led to accumulation of more oleic acid (C18:1) and less linoleic acid (C18:2) making the peanut oil with high O/L ratio.

Oleic (C18:1) (monounsaturated) and linoleic acids (C18:2) (diunsaturated) together account for 80% of total oil content in peanut [7]. The improved shelf life of peanut oil is because of multifold (10 fold) higher anti-oxidative stability in presence of high oleic acid (C18:1) as compared to presence of high linoleic acid [8]. Consuming peanut products using the seed containing high oleic acid has several health benefits such as reduction of serum cholesterol level, suppression of tumorigenesis and amelioration of inflammatory diseases [9,10]. Both the fatty acids i.e., oleic acid (C18:1) and linoleic acid (C18:2) are known to lower the level of bad cholesterol (low-density lipoprotein, LDL). The oleic acid (C18:1) provides more advantage over linoleic acid (C18:2) by not affecting good cholesterol (high-density lipoprotein, HDL) levels [11]. This is because the saturated fatty acids are known to be hyper-cholesterolemic, polyunsaturated fatty acids are hypo-cholesterolemic while monounsaturated fatty acids are known to be neutral [12]. Moreover, oil with higher unsaturated fatty acids allows heating without smoking at high temperatures, which leads to faster cooking and less oil absorption by the cooked food [13]. In addition, higher concentration of polyunsaturated fatty acids (PUFA) such as linoleic and linolenic acids makes the cooked product more susceptible to rancidity and decreases flavor rapidly along with shortening the shelf life. Therefore, breeding peanuts with high O/L ratio along with high oil content or low oil content will have direct impacts on profitability of growers, peanut industry and consumer preferences such as low fat foods.

The challenge before the breeding program is to target oil content and O/L ratio in addition to yield enhancement [14-16]. The effort led to identification of the first mutant (F435) with high oleic acid (C18:1) at the University of Florida. The difference in O/L ratio obtained between existing peanut germplasm is very low (only 1.0 to 2.5 O/L ratio) as compared to the high oleic mutant (up to 40.0 O/L ratio) [17]. This mutant line was then utilized for development of a series of breeding lines with high oleic acid [18,19].

Oil content in peanut seeds is a complex trait controlled by a number of genes with significant environmental influences. Molecular markers have been used to discover quantitative trait locus (QTL) or chromosomal regions associated with seed oil in other oil crops [20]. The identification of markers or QTL for peanut oil will have potential application in molecular breeding, which could facilitate the development of high or low oil content peanuts with the high oleic trait. In the present study, two recombinant inbred line (RIL) populations used to address the following objectives: (1) to improve the genetic linkage maps developed by Qin et al. [21], (2) to identify QTL for oil content and quality, (3) to map the *FAD2* on the peanut genome and (4) to determine the effects of *FAD2* genes on oil content and oil quality.

## Results

### Development of improved genetic maps

Two recombinant inbred line (RIL) populations namely S-population (SunOleic 97R × NC94022) and T-population (Tifrunner × GT-C20) were used to construct genetic maps with 172 and 239 loci, respectively [21]. The present study further improved these two genetic maps to 206 and 378 marker loci for the S-population and the T-population (Additional files 1 and 2), respectively. For the S-population, 206 mapped loci were distributed on 20 linkage groups (LGs) covering a total genome distance of 1780.6 cM and achieved a map density of 9.6 cM/loci. Similarly for the T-population, 378 loci were mapped onto 20 linkage groups covering a total map distance of 2487.4 cM with a map density of 7.0 cM/loci (Table 1, Additional files 3 and 4). The number of mapped marker loci per LG were ranged from 3 loci (a02, a08 and b05) to 18 loci (a03) in the S-population while 10 loci (b03) to 35 loci (a04) in the T-population. Similarly, the individual length of LGs ranged from 29.9 cM (a02) to 244.3 cM (b09) in the S-population, and 52.4 cM (a07) to 200.9 cM (a08) in the T-population. Of the total 206 mapped loci in the S-population, 110 loci could be mapped on the A sub-genome with a total map distance of 799.4 cM and 96 loci were mapped on the B sub-genome with a map distance of 981.2 cM. Similarly in the T-population, 225 and 153 loci were mapped on the A and the B sub-genome, resulting in the total map distance of 1242.1 and 1245.2 cM, respectively.

**Table 1:** **Important features of the genetic maps constructed for the S-population and the T-population**

**S No**	**Linkage group**	**S-population**			**T-population**		
		**Total map distance**	**Mapped loci**	**Map density**	**Total map distance**	**Mapped loci**	**Map density**
		**(cM)**		**(cM/loci)**	**(cM)**		**(cM/loci)**
A sub-genome linkage groups							
1	a01	61.1	13	4.7	179.1	22	8.1
2	a02	29.9	3	10.0	117.5	12	9.8
3	a03	66.5	18	3.7	150.2	31	4.8
4	a04	103.9	8	13.0	121.7	35	3.5
5	a05	150.4	17	8.8	102.5	24	4.3
6	a06	99.2	6	16.5	158.7	26	6.1
7	a07	118.1	17	6.9	52.4	13	4.0
8	a08	28.5	3	9.5	200.9	28	7.2
9	a09	74.4	17	4.4	77.0	18	4.5
10	a10	67.4	8	8.4	82.1	16	5.1
B sub-genome linkage groups							
11	b01	68.4	12	5.7	87.0	13	6.7
12	b02	69.7	8	8.7	115.8	20	5.3
13	b03	156.7	12	13.1	120.1	10	12.0
14	b04	91.1	13	7.0	190.2	21	9.1
15	b05	62.3	3	20.8	154.8	13	11.9
16	b06	39.0	4	9.8	83.3	13	6.4
17	b07	157.6	12	13.1	134.0	18	7.4
18	b08	54.8	11	5.0	101.3	16	6.3
19	b09	244.3	15	16.3	124.4	13	9.6
20	b10	37.3	6	6.2	134.4	16	8.4
Total /mean		1780.6	206	9.6	2487.4	378	7.0

### Identification of main-effect QTLs (M-QTLs) by QTLCartographer

**Figure 1:**
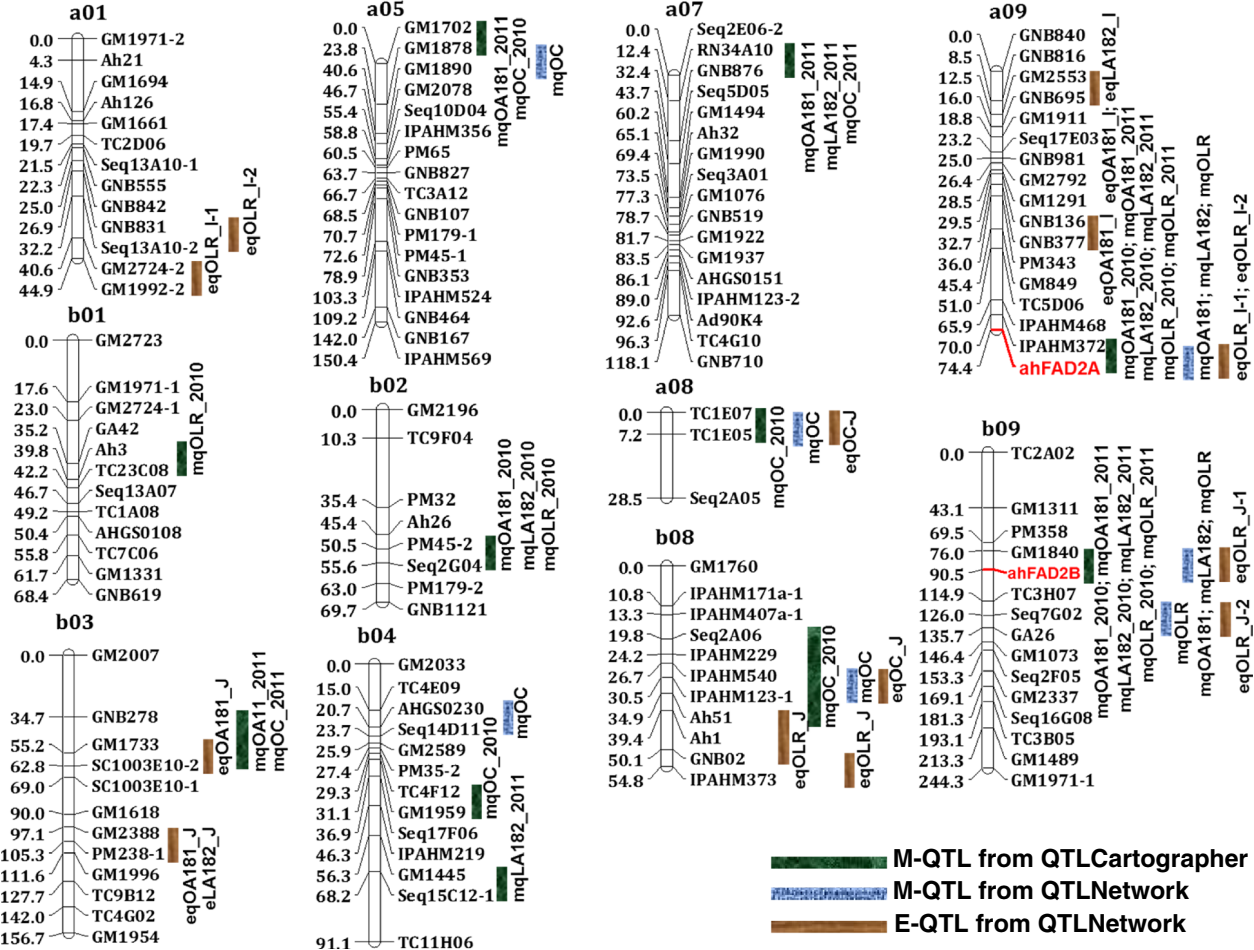
**Genetic map of the S-population showing main-effect (M-QTLs) and epistatic (E-QTLs) QTLs for oil content and quality traits.** This figure shows positions of 38 M-QTLs detected by QTLCartographer and QTLNetwork while eight E-QTLs detected by QTLNetwork on peanut genome.

**Figure 2:**
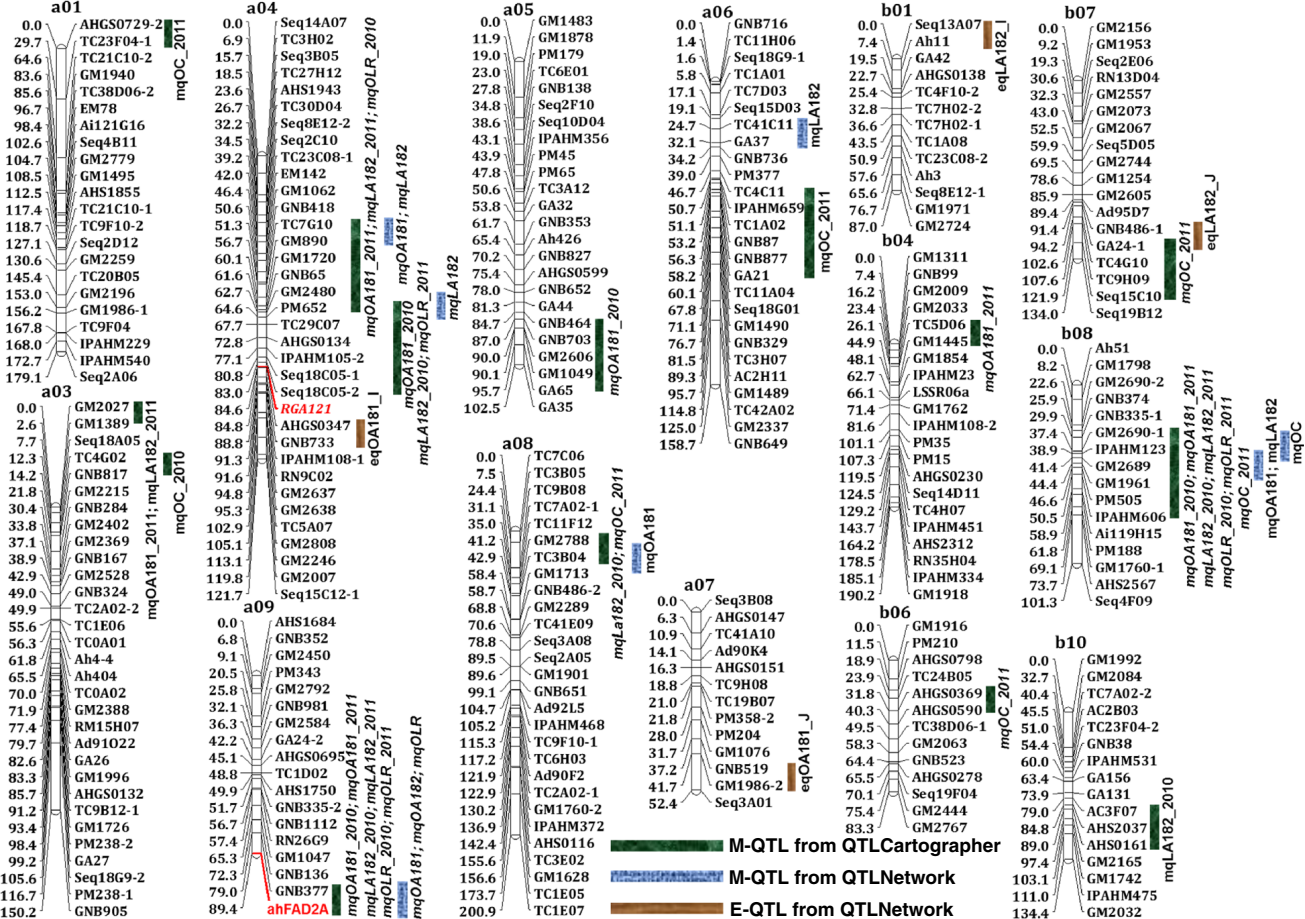
**Genetic map of the T-population showing main-effect (M-QTLs) and epistatic (E-QTLs) QTLs for oil content and quality traits.** This figure shows positions of 40 M-QTLs detected by QTLCartographer and QTLNetwork while two E-QTLs detected by QTLNetwork on peanut genome.

Phenotypic data obtained for two seasons for oil content and quality traits were analyzed together with genotypic data for both the populations using Windows QTLCartographer. QTL analysis resulted in identification of a total of 27 (S-population) and 29 (T-population) M-QTLs for oil content and quality traits with PVE ranging up to 42.33% and 28.98%, respectively (Table 2, Figures 1 and 2). The highest logarithm of odds (LOD) value could be observed for O/L ratio (up to 118.87) in the S-population and for linoleic acid (C18:2) (up to 15.8) in the T-population. Further, the LOD value ranged from 2.85 to 9.27 and 2.53 to 8.00 for oil content in the S- and the T-population, respectively (Table 2). Of the 27 and 29 QTLs identified for oil content and quality traits in S- and T-population, seven and six QTLs were major QTLs (>10% PVE), respectively (Additional files 5 and 6).

**Table 2:** **Summary of main-effect QTLs (M-QTLs) identified by QTLCartographer in the S-population and the T-population**

**Traits**	**S-population**				**T-population**			
	**QTLs identified**	**LOD value range**	**Phenotypic variance range (%)**	**Additive effect range (a0)**	**QTLs identified**	**LOD value range**	**Phenotypic variance range (%)**	**Additive effect range (a0)**
Oleic acid (C18:1)	8	2.50-33.09	1.59-27.54	5.04 to (-) 12.758	9	2.52-15.44	3.63-28.98	4.095 to (-) 2.12
Linoleic acid (C18:2)	7	2.54-32.41	1.46-28.22	10.878 to (-) 2.717	9	3.72-15.8	3.91-25.49	1.873 to (-) 3. 20
Oleic/linoleic acid ratio (OLR)	6	2.53-118.87	1.04-42.33	1.13 to (-) 12.29	5	3.78-9.82	5.70-14.90	0.82 to (-) 0.221
Oil content (OC)	6	2.85-9.27	3.07-10.23	3.53 to (-) 4.44	9	2.53-8.00	3.93-14.07	0.858 to (-) 0.601

For oleic acid (C18:1), a total of eight M-QTLs in the S-population and nine M-QTLs in the T-population were identified with PVE up to 27.54% and 28.98%, respectively (Table 2). The *ahFAD2A* (7.76% and 8.40%) and *ahFAD2B* (27.54% and 25.54%) showed consistent high contributions in both years in the S-population, while *ahFAD2A* (28.98% and 12.13%) contributed in similar way in the T-population. The *ahFAD2A* and *ahFAD2B* genes contributed for high oleic acid (C18:1) and the contributing mutant allele came from the ‘SunOleic 97R’ parent in the S-population. Similarly in the T-population, the *ahFAD2A* gene contributed for high oleic acid (C18:1) and the contributing mutant allele came from ‘Tifrunner’. In terms of consistency of the QTLs, the QTLs identified in both years (2010 and 2011) were considered as “consistent” QTLs. Two consistent QTLs namely IPAHM372-*ahFAD2A* and GM1840-*ahFAD2B* in the S-population (Additional file 5) and two consistent QTLs namely GNB377-*ahFAD2A* and GM2690-1-IPAHM606 were identified for oleic acid (C18:1) in T-population (Additional file 6).

For linoleic acid (C18:2), a total of seven and nine M-QTLs were detected in the S- and the T-population with PVE up to 28.22% and 25.49%, respectively (Table 2). The *ahFAD2A* (7.97% and 5.76%) and *ahFAD2B* (28.22% and 22.96%) genes showed consistent high contribution in both years in the S-population and, in similar way, *ahFAD2A* (25.49% and 11.98%) contributed in the T-population. In contrast to oleic acid (C18:1), the contributing alleles of *ahFAD2A* and *ahFAD2B* genes for high linoleic acid (C18:2) came from the parent ‘NC94022’ of the S-population while contributing allele for *ahFAD2A* came from the parent ‘GT-C20’ of the T-population. In terms of consistency of the QTLs, two consistent QTL regions namely IPAHM372-*ahFAD2A* and GM1840-*ahFAD2B* were identified for linoleic acid (C18:2) in the S-population (Additional file 5). Similarly in the T-population, two consistent QTLs namely GNB377-*ahFAD2A* and GM2690-1-IPAHM606 were identified for linoleic acid (C18:2) (Additional file 6).

A total of six and five M-QTLs were detected for O/L ratio in the S- and the T-population with PVE up to 42.33% and 14.90%, respectively (Table 2). Similar to oleic acid (C18:1) and linoleic acid (C18:2), *ahFAD2A* (4.16% and 3.42%) and *ahFAD2B* (39.71% and 42.33%) genes showed consistent high contribution in both the years in the S-population while *ahFAD2A* (14.90% and 6.08%) showed in the T-population. The results for O/L ratio were similar to oleic acid (C18:1) and in contrast to linoleic acid (C18:2). The mutant alleles of *ahFAD2A* and *ahFAD2B* genes present in ‘SunOleic 97R’ parent of the S-population contributed for high oleic acid (C18:1) while mutant allele of *ahFAD2A* gene present in ‘Tifrunner’ contributed for O/L ratio in the T-population. In terms of consistency of the QTLs for O/L ratio, only two consistent QTLs namely IPAHM372-*ahFAD2A* and GM1840-*ahFAD2B* were identified in the S-population (Additional file 5). The above two consistent QTLs harboured well known *ahFAD2A* and *ahFAD2B* genes on LG a09 and b09, respectively (Figure 1). In the T-population, one consistent QTL namely GNB377-*ahFAD2A* could be identified for O/L ratio (Additional file 6).

**Figure 3:**
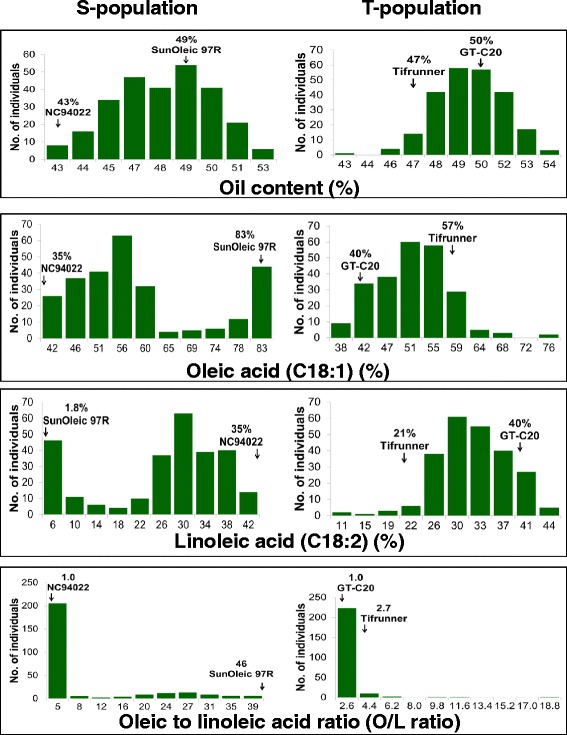
**Distribution of oil content, oleic acid, linoleic acid and oleic/linoleic acid ratio in the S- and T- populations.** The x-axis shows the range of percentage of average of two years of oil content, oleic acid, linoleic acid and oleic/linoleic acid ratio and the y-axis represents the number of individuals in each RIL population.

The distribution of total oil content in the S- and the T-population was normal (Figure 3). For oil content, a total of six and seven M-QTLs were identified in the S- and the T-population with PVE up to 10.23% and 14.07%, respectively (Table 2). It was interesting to note that no consistent QTL could be identified for oil content in either of the populations (Additional files 5 and 6).

In addition to the identification of consistent QTLs for a single trait on a particular genomic region, such QTLs for multiple traits were also found on the same genomic regions. Interestingly, two QTLs (IPAHM372-*ahFAD2A* and GM1840-*ahFAD2B)* on a09 and b09 in the S-population and one QTL (GNB377-*ahFAD2A)* on a09 had two consistent QTLs each for oleic acid (C18:1), linoleic acid (C18:2) and O/L ratio (Table 2, Additional files 5 and 6, Figures 1 and 2). In addition to these QTLs, the other QTLs which were found to control more than one trait are GM1702-GM1878 (oleic acid and oil content) and RN34A10-GNB876 (oleic acid, linoleic acid and oil content) in the S-population (Additional file 5) while GM2690-1-IPAHM606 (oleic acid, linoleic acid, O/L ratio and oil content), PM652-Seq18C05-2 (oleic acid, linoleic acid and O/L ratio), TC7G10-PM652 (oleic acid, linoleic acid and O/L ratio), and GM2788-TC3B04 (linoleic acid and oil content) in the T-population (Additional file 6).

### Identification of main-effect QTLs (M-QTLs) by QTLNetwork

Analysis using QTLNetwork resulted in identification of a total of 11 M-QTLs each for the S- and T-population for all the four oil traits and PVE ranged from 0.25 to 25.52% and 0.46 to 29.13%, respectively (Table 3, Additional files 7 and 8, Figures 1 and 2). Of the total QTLs identified in both the populations, four QTLs in the S-population and two QTLs in the T-population had major phenotypic effect (>10% PVE) (Additional files 7 and 8).

**Table 3:** **Summary of main-effect QTLs (M-QTLs) identified by QTLNetwork in the S-population and the T-population**

**Traits**	**S-population**				**T-population**			
	**QTLs identified**	**P-value range**	**Phenotypic variance range (%)**	**Additive effect range (a0)**	**QTLs identified**	**P-value range**	**Phenotypic variance range (%)**	**Additive effect range (a0)**
Oleic acid (C18:1)	2	0	8.72-14.18	(-) 4.44 to (-) 5.91	4	0.00	0.46-29.13	4.49 to (-) 1.63
Linoleic acid (C18:2)	2	0.00	8.36-13.83	3.42 to 4.91	5	0.00- 1.6xE-5	0.86-26.64	1.02 to (-) 3.70
Oleic/linoleic acid ratio (OLR)	3	0.0 to 3.1xE-5	0.25-10.82	1.47 to (-) 3.90	1	0.00	5.19	0.422
Oil content (OC)	4	0.00	4.79 - 25.52	0.533 to (-) 1.465	1	0.00	6.70	0.546

For oleic acid (C18:1), two M-QTLs in the S-population while four M-QTLs in the T-population were identified with PVE up to 14.18% and 29.13%, respectively. Only one major M-QTL each could be detected for oleic acid (C18:1) in the S-population (GM1840-*ahFAD2B* with 14.18% PVE) and T-population (GNB377-*ahFAD2A* with 29.13% PVE). Additive effect for *ahFAD2A* and *ahFAD2B* genes indicated that the contribution for high oleic acid (C18:1) came from the ‘SunOleic 97R’ parent in the S-population and for *ahFAD2A* gene from ‘Tifrunner’ in the T-population. Similarly, two and five M-QTLs were detected for linoleic acid (C18:2) in the S- and T-population with PVE up to 13.83% and 26.64%, respectively. Only one major M-QTL could be detected for linoleic acid (C18:2) in the S-population (GM1840-*ahFAD2B* with 13.83% PVE) and T-population (GNB377-*ahFAD2A* with 26.64% PVE). Additive effect for *ahFAD2A* and *ahFAD2B* indicated that the contribution for high linoleic acid (C18:2) came from the parent ‘NC94022’ in the S-population and for *ahFAD2A* gene from ‘GT-C20’ in the T-population. Three M-QTLs with PVE up to 10.82% in the S-population and one M-QTL with PVE up to 5.19% were detected for O/L ratio. Only one major M-QTL could be detected for O/L ratio in the S-population (GM1840-*ahFAD2B* with 10.82% PVE) while no major M-QTL was detected in the T-population (Additional files 7 and 8). For oil content, a total of four M-QTLs in the S-population and one M-QTL in the T-population were identified with PVE up to 25.52% and 6.7%, respectively. Only one major M-QTL could be detected for oil content in the S-population (GM1878-GM1890 with 25.52% PVE) while there was no major M-QTL identified in the T-population. For this major QTL (25.52% PVE), the allele from the parent ‘SunOleic 97R’ contributed towards high oil content while allele from the parent ‘NC94022’ contributed towards low oil content. The same QTL was also detected by QTLCartographer.

### Identification of epistatic effect QTLs (E-QTLs) by QTLNetwork

**Figure 4:**
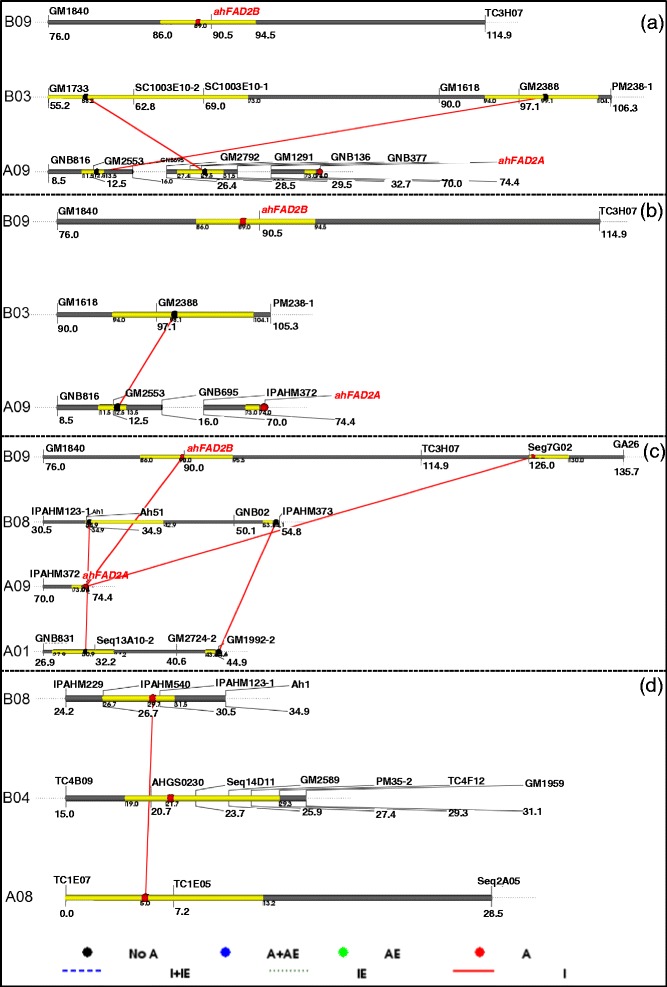
**Epistatic interaction identified by QTL Network for oil quality traits.** Figure shows epistatic interaction for **(a)** oil content, **(b)** oleic acid, **(c)** linoleic acid, and **(d)** oleic/linoleic (O/L) ratio. The *black ball* represents epistatic QTLs without individual effect; the *blue ball* represents additive × (additive × environment) interaction; the *red ball* indicates epistatic QTLs with direct individual effect while interacting loci are shown by *red colored bars.*

QTL analysis using QTLNetwork for oil content and quality traits resulted in identification of ten E-QTLs (eight in the S-population and two in the T-population) with only two-locus interactions (Figure 4, Table 4, Additional file 9). The PVE for E-QTLs detected in the S-population ranged from 0.13 to 3.1% and additive effect due to interaction of both the loci varied from 3.08 to -1.06. Similarly, the PVE% for E-QTLs detected in the T-population ranged from 1.69 to 2.9% and additive effect due to interaction of both the loci varied from 1.12 to 1.17%. *FAD2* genes/alleles were found to be involved in two out of the ten interactions and both interactions involved linoleic acid (C18:2) in the S-population. The other QTLs which had appeared in more than one interaction include GM2553-GNB695 and GM2388-PM238-1 (Additional file 9). Three two-locus interactions were identified for oleic acid with the PVE ranging from 1.6 to 2.89% i.e., two in the S-population and one in the T-population. One two-locus interaction was identified in each population for linoleic acid (C18:2) with PVE ranging from 2.82 to 2.90% (Table 4, Additional file 9). No E-QTL could be identified for O/L ratio and oil content in the T-population, while four E-QTLs could be identified for O/L ratio (PVE up to 3.1%) and a single E-QTL for oil content with PVE of 0.88%.

**Table 4:** **Summary of epistatic QTLs (E-QTLs) identified by QTLNetwork in the S-population and the T-population**

**Traits**	**QTLs identified**	**PVE range (%)**	**AA range**	**SE range**	**P-value range**
S-population					
Oleic acid (C18:1)	2	1.6-2.83	3.08 to (-) 2.58	0.510 - 0.514	0.00
Linoleic acid (C18:2)	1	2.82	−2.0905	0.4311	1xE-6
Oleic/linoleic acid ratio (OLR)	4	0.13-3.1	2.11 to (-) 1.43	0.378-0.399	0.00 to 3xE-4
Oil content (OC)	1	0.88	−0.2623	0.085	0.002041
T-population					
Oleic acid (C18:1)	1	1.69	1.1219	0.2896	1xE-4
Linoleic acid (C18:2)	1	2.9	1.1695	0.2292	0

AA: The estimated additive effect; SE: The standard error of estimated or predicted QTL effect and P-value.

## Discussion

### Trait importance and development of RIL mapping populations

Oleic acid (C18:1) is a monounsaturated fatty acid while linoleic acid (C18:2) is a polyunsaturated fatty acid and both together make upto 80% of the oil composition. The desirability of high oleic acid (C18:1) lies in its good property of providing longer shelf life due to ten-fold higher anti-oxidative stability compared to linoleic acid (C18:2). Besides longer shelf life, it also plays an important role to human health by decreasing blood LDL levels, suppressing tumorigenesis and ameliorating inflammatory diseases [8,9,22]. Enhancing or lowering oil content is an important breeding objective in majority of the breeding programs of the world. In this context, the present study was done to identify QTLs/linked markers associated with oil content to deploy them after validation in developing improved genotypes with desired level of oil content. In addition to the oil content, improving the peanut oil quality is another major breeding objective after pod yield and oil content. Although gene-based markers are available for both the mutant *FAD2* genes (*ahFAD2A* and *ahFAD2B*), their location on the peanut genome and their relative contribution towards oil quality are not known. Therefore, two RIL mapping populations were developed and used in this study to generate information to meet the research needs. The S-population segregated for both the *FAD2* genes (*ahFAD2A* and *ahFAD2B*) while the T-population segregated for only one *FAD2* gene (*ahFAD2A*).

It is well known that small population size (100-200 lines) has adverse effect on the accuracy in identifying QTL positions and estimating QTL effects [23,24]. Two large RIL populations with 352 (S-population) and 248 (T-population) individuals were developed and used to phenotype for oil content and quality traits in two successive years (2010 and 2011). These two populations were then used for locating the position of QTLs and *FAD2* genes on the peanut genome, and identification of associated markers for oil content and quality traits.

### Development of improved genetic maps

Both genetic maps were enriched with additional polymorphic markers i.e., from 172 loci to 206 loci for the S-population and from 236 loci to 378 loci for the T-population in comparison with the earlier maps by Qin et al. [21]. In both the populations, comparatively higher number of loci could be mapped in the A sub-genome (110 loci in the S-population and 224 loci in the T-population) than the B sub-genome (94 loci in the S-population and 153 loci in the T-population). The genome coverage of T-population was higher (2487.4 cM) than the S-population (1780.6 cM). The map features of these two populations indicated that the A sub-genome is more diverse than the B sub-genome.

So far only seven genetic maps based on RIL populations have been reported in peanut. Individual genetic maps were constructed for the S- and T-population earlier with 172 (920.7 cM) and 236 (1,213.4 cM) marker loci, respectively [21]. The other five genetic maps based on RILs included TAG 24 × ICGV 86031 (291 loci, 1,785.4 cM, [25,26]), ICGS 76 × CSMG 84-1 (119, loci, 2,208.2 cM, [27]), ICGS 44 × ICGS 76 (82 loci, 831.4 cM, [27]), TAG 24 × GPBD 4 (188 SSR loci, 1,922.4 cM, [28] ) and TG 26 × GPBD 4 (181 SSR loci, 1,963 cM, [28]). Thus, the current map of the T-population possesses the highest number (378) of marker loci among all the genetic maps constructed so far using RIL population.

### Identification of QTLs for oil content and quality traits

Total PVE of a complex trait results from the presence of multiple QTLs as well as their interactions (QTL to QTL and QTL to environment). Hence, in the present study two genetic softwares were used for identification of M-QTLs (QTLCartographer and QTLNetwork) and E-QTLs (QTLNetwork). QTL analysis resulted in identification of a total of 38 (27 by QTLCartographer and 11 by QTLNetwork) M-QTLs in the S-population and 40 (29 by QTLCartographer and 11 by QTLNetwork) M-QTLs in the T-population. The PVE ranged from 0.24 to 42.33% in the S-population and from 0.46 to 28.98% in the T-population. In case of E-QTLs, ten E-QTLs (eight in the S-population and two in the T-population) were detected with PVE ranging from 0.13-3.1% and additive effect ranging from 3.08 to -1.06. It was interesting to note that *FAD2* genes/alleles were found to be involved in two out of ten interactions and both involved linoleic acid in the S-population.

It was interesting that there was three-fold difference in detection of QTLs by both the software used in this study. The difference in number of QTLs detected is due to the variation in the algorithm of the software. The CIM of the QTLCartographer fits parameter to target QTL in one interval and simultaneously fits partial regression coefficients for background markers in order to account variance due to non-target QTL. It allows this software to consider various gene actions (additive and dominance) and QTL by environment interactions and close linkage. On the other hand, the CIM of the QTLNetwork is based on the mixed-model method.

As expected *FAD2* genes controlled three oil quality traits (oleic acid, linoleic acid and O/L ratio) but had no effect on oil content. Results clearly showed that the contribution of *ahFAD2B* to PVE for oleic acid (C18:1), linoleic acid (C18:2) and O/L ratio were always higher than the contribution of the *ahFAD2A* gene. The QTL ‘TC6H03-TC11A04’ had been reported earlier for significant contribution to oleic acid (9.70% PVE), linoleic acid (9.00% PVE) and O/L ratio (6.80% PVE) [29]. The low PVE reported by Sarvamangala et al. [29] may have been due to the low level of divergence among the parental genotypes for oil quality traits and the lack of sufficient marker loci representing the peanut genome. More recently, 25 marker-trait associations (MTAs) for oil content (5.84-40.37% PV), two MTAs for oleic acid (16.42% PV) and 22 MTAs for O/L ratio (13.67-47.45% PV) were identified in a comprehensive genome-wide association studies (GWAS) [30]. Of the four associated markers TC4G02, Seq7G02, TC11A04 and Seq3B05 for oil content [30], the marker TC11A04 was also found associated with oil quality traits in the present study as well as the previous study [29].

No detailed studies on *FAD2* genes towards their role in controlling oil quality and content have been conducted in peanut and hence no literature is available to draw comparisons. Nevertheless, similar studies were conducted in other crops such as rapeseed (*Brassica napus*) [31] and soybean (*Glycine max*) [32]. Similar to peanut, two *FAD2* genes are reported to be present in rapeseed (*B. napus*) on two different genomes i.e., the A-genome and the C-genome. However in soybean, two oleate desaturase genes (*FAD2-1A* and *FAD2-1B*) and three linoleate desaturase genes (*FAD3A, FAD3B* and *FAD3C*) were identified and unambiguous chromosomal positions were assigned [32]. It was clearly indicated in rapeseed that the QTL for oleic acid had a negative effect on linoleic acid [31] which is also been found in the present study. We have clearly observed that *ahFAD2A* and *ahFAD2B* mutant alleles increased the quantity of oleic acid (C18:1) and decreased the production of linoleic acid (C18:2) which resulted in high O/L ratio. Thus, these studies provide genetic evidence that the gene products of these *FAD2* alleles catalyze the conversion of oleic acid (C18:1) to linoleic acid (C18:2).

### Consistent M-QTLs for improving oil content and quality traits

Realizing the practical importance of consistent QTLs over seasons, *ahFAD2A* and *ahFAD2B* genes showed a consistent high contribution in the S-population and *ahFAD2A* in the T-population for oleic acid (C18:1), linoleic acid (C18:2) and O/L ratio. These two consistent QTLs namely IPAHM372-*ahFAD2A* and GM1840-*ahFAD2B* in the S-population while GNB377-*ahFAD2A* in the T-population controlled three oil quality traits namely oleic acid (C18:1), linoleic acid (C18:2), and O/L ratio. It was interesting to note that the additive effect for *ahFAD2A* and *ahFAD2B* indicated that the contribution for high oleic acid and O/L ratio came from the ‘SunOleic 97R’ parent in the S-population while for *ahFAD2A* gene from ‘Tifrunner’ in the T-population. In contrast to oleic acid (C18:1), additive effect for *ahFAD2A* and *ahFAD2B* indicated that the contribution for high linoleic acid (C18:2) came from the ‘NC94022’ parent in the S-population and for *ahFAD2A* gene from ‘GT-C20’ in the T-population.

Among consistent QTLs, two consistent QTL regions were identified, each for oleic acid (C18:1) and linoleic acid (C18:2) in both the populations. In case of O/L ratio, two consistent QTL regions in the S-population and one consistent QTL region were identified in the T-population. For oil content, no consistent QTL could be identified in either of the populations which shows the complexity of the trait and extent of environmental influence. In addition to the above consistent QTLs, the two other QTLs controlling more than one trait were also identified in the S-population and four in the T-population. It is noted that one *RGA-121* marker was mapped on a04 with linkage to oil quality traits (Figure 2, Additional file 6) in the T-population, was also reported to be linked to disease resistance QTLs [33]. The consistent QTLs identified in this study provided confidence on these QTLs and their role towards controlling these traits. Such consistent QTLs have earlier been identified for foliar fungal diseases and were also successfully deployed in genomics-assisted breeding for improving rust resistance [34]. Therefore, the markers underlying these consistent QTLs are of great importance and may be deployed after validation in improving oil content and oil quality traits through genomics-assisted breeding.

### Relative contribution of mutant alleles towards oil quality traits

The current general understanding is that genotypes possessing both the mutant alleles (*ahFAD2A* and *ahFAD2B*) will produce higher oleic acid (C18:1) and reduced linoleic acid (C18:2). The mutant allele *ahFAD2A* is widely available in the U.S. peanut germplasm collection and in elite genotypes but mutant allele *ahFAD2B* is not available in the U.S. germplasm collection [35]. The mutant allele *ahFAD2B* is present in selected genotypes such as SunOleic 95R, SunOleic 97R, most of which trace their pedigrees to F435 (except Flavorunner 458). There are no systematic studies on estimating phenotypic contribution of QTLs and these two mutant alleles towards oil quality traits but there are surveys and studies recently on *FAD2* genes effect on fatty acid profiles and oil content [36-39]. This fact also raises a question that what makes the two mutant alleles to produce more oleic acid (and less linoleic acid) when both mutant alleles are present together and less oleic acid (and high linoleic acid) when either of the mutant alleles are present separately (Table 5). Further, the involvement of other factors in influencing the production of oleic acid should be very possible. Therefore, more information on this aspect needs to be generated for improving further understanding of the genetic control and pathway functionality for fatty acid synthesis in peanut.

**Table 5:** **Phenotypic value in percentage of the oil quality traits in RILs by genotypes of** ***ahFAD2A*** **and** ***ahFAD2B*** **genes in the S-population and the T-population**

**Quality traits**	**S-Population**				**T-population**	
	**AABB (66)**	**AAbb (51)**	**aaBB (65)**	**aabb (60)**	**AABB (92)**	**aaBB (130)**
Oleic acid (C18:1)	46.52	57.03	55.58	70.23	44.2	52.56
Linoleic acid (C18:2)	32.50	23.79	25.03	12.72	34.2	27.44
Oleic/linoleic acid ratio (OLR)	2.17	5.09	4.50	17.68	1.35	2.22

AA: wild A sub-genome allele for *ahFAD2A* gene in homozygous condition, aa: mutant A sub-genome allele for *ahFAD2A* gene in homozygous condition, Aa: *ahFAD2A* gene in heterozygous condition in A sub-genome, BB: wild B sub-genome allele for *ahFAD2B* gene in homozygous condition, bb: mutant B sub-genome allele for *ahFAD2B* gene in homozygous condition, Bb: *ahFAD2B* gene in heterozygous condition in B sub-genome. The number in parentheses is the number of RILs with that specific genotype.

## Conclusion

Oil content and quality traits have high impact on peanut markets due to profitability and consumers preference for several health benefits. The *FAD2* genes are known to control some of these traits and their position on the peanut genome and their contributions towards total phenotypic variance for these quality traits were unknown. Two RIL mapping populations were used for identification of QTL positions and estimating QTL effects.

This study reports the development of two improved genetic maps and identification of 78 M-QTLs and 10 E-QTLs for oil content and three oil quality traits (oleic acid, linoleic acid and O/L ratio). The *ahFAD2A* and *ahFAD2B* genes were mapped to the homeologous linkage groups of A (a09) and B sub-genome (b09). The results indicated that the contribution of both the mutant alleles together was much higher than the cumulative individual effect of *FAD2* genes. Further, the QTL analysis always detected higher PVE for *ahFAD2B* for oleic acid (C18:1), linoleic acid (C18:2) and O/L ratio than the *ahFAD2A* genes. This study not only estimated phenotypic effect of both the *FAD2* genes for (C18:1), linoleic acid (C18:2) and O/L ratio but also identified additional QTLs controlling these quality traits. By increasing the proportion of oleic acid in peanut oil, at the expense of linoleic acid, the oxidative stability can be increased in addition to the health benefits. The information generated through this study should be very useful for marker-assisted development of improved peanut varieties with desired oil content and quality traits.

## Methods

### Development of mapping populations

Two recombinant inbred line (RIL) populations derived from the crosses ‘SunOleic 97R’ [19] × ‘NC94022’ (S-population), and ‘Tifrunner’ [40] × ‘GT-C20’ (T-population) were developed following single seed decent (SSD) method at Crop Protection and Management Research Unit, USDA-ARS, Tifton, USA. The genotype ‘SunOleic 97R’ was developed from the cross ‘SunOleic 95R’ × ‘Sunrunner’ , and ‘NC94022’ is a breeding line derived from the cross ‘N91026E’ × ‘PI 576638’. The female parent of the T-population, ‘Tifrunner’, is a runner market-type cultivar and the male parent, ‘GT-C20’ , is a Spanish-type breeding line. The S-population and the T-population had 352 and 248 individuals, respectively and were used for multiseason phenotyping for oil content and three oil quality (oleic acid, linoleic acid and O/L ratio) traits.

### Phenotyping of mapping populations

Full sets of the S- and T-population along with parental genotypes were grown in three replications during 2010 (F_7_ generation) and 2011 (F_8_ generation) at the Bellflower Farm, Tifton, GA. Recommended agronomic and management practices were followed to grow a healthy crop. Harvested pods from all the replications of RIL lines were properly dried, packed and sent to USDA-ARS, Griffin (USA) for chemical analysis of oil content, oleic acid (C18:1) and linoleic acid (C18:2). The O/L ratio was calculated using the values of oleic acid (C18:1) and linoleic acid (C18:2).

*Oil content:* A Maran Pulse nuclear magnetic resonance (NMR, Resonance Instruments, Whitney Oxfordshire, UK) was used to determine the oil content in percentage. The NMR calculated oil% and H_2_O% in the sample. Total 5-10 g of whole mature seeds were weighed and analyzed for each of two subsamples per entry. Oil percentage was calculated and determined on a basis of zero percent water content in seed by using the formula [oil% × 100/(100 – H_2_O% × 100)].

*Oleic* (C18:1) *and linoleic* (C18:2) *acids:* Three to five seeds were ground to a fine powder in a coffee bean grinder. Approximately 150 mg of ground powder was transferred into a 16 × 100 mm disposable test tube, and 5.0 ml of n-heptane (Fisher Scientific) was added to extract the oil. For conversion of fatty acids to methyl esters, 500 μl of 0.5 N sodium methoxide (NaOCH_3_) in methanol solution was added to the test tube and mixed with the sample. After 2 hours, 7.0 ml of distilled water was added to separate the organic layer from the aqueous layer and seed residue (45 min). An aliquot of the organic layer (1.5 ml) containing the methyl esters was transferred to a 2.0 ml autosampler vial for GC analysis. Fatty acid composition was determined using an Agilent 7890A gas chromatograph (GC) equipped with a flame ionization detector (FID) and an autosampler. A fatty acid methyl ester (FAME) standard mix RM-3 (purchased from Sigma) was used to establish peak retention times. Peak separation was performed on a DB-225 capillary column (15 m × 0.25 mm i.e. with a 0.25 μm film) from Agilent Technologies. The carrier gas was helium set to a flow rate of ~1.0 ml/min. One μl of sample was injected at a 60:1 split ratio onto the column maintained isothermally at 210°C. The inlet and detector were set to 280°C to 300°C, respectively. Total run time for each sample was 12 minutes. Oleic (C18:1) and linoleic (C18:2) acid composition was determined by identifying and calculating relative peak areas.

### DNA extraction and genotyping of genetic material

DNA was extracted from fresh leaves of the parental genotypes and the RILs as described in Qin et al. [21]. After assessing the quality and quantity of isolated genomic DNA in Nano Drop-1000 spectrophotometer, PCR reactions were carried out in 15 μl reaction volumes using thermal cycler (PTC-225 DNA Engine Tetrad Peltier, MJ Research, USA and DNA Engine Tetrad 2 Peltier, BioRad Laboratories, USA). The master mix was prepared using 0.5 μM of each primer, 25 ng genomic DNA, 10X PCR buffer, 1.5 mM MgCl, 0.2 mM of dNTPs and 0.5 U of Taq polymerase. PCR profiles and band scoring was done as explained in Qin et al. [21]. A total of 230 and 402 polymorphic markers were identified for the S- and the T-population, respectively. Genotyping data for 215 SSR loci in the S-population and 390 SSR loci in the T-population were generated on the full sets of RILs. The information on source of the markers and names used in Qin et al. [21] and present study has been provided in Additional file 10.

### Construction of improved genetic maps

Genotyping data obtained for all the polymorphic marker loci were scored as “a” and “b” to use in the construction of an improved genetic map using JoinMap® version 4. Genotyping data were first analysed for segregation distortion for each marker loci to calculate chi-square values using a “locus genotype frequency” function against the expected 1:1 ratio. Due to segregation distortion for some SSR loci, initially a framework genetic map was prepared with normally segregating markers at LOD of 4.0 with a minimum recombination threshold of 40%. Marker loci were placed into respective linkage groups (LG) using the command “LOD groupings” and “create groups for mapping”. The Kosambi map function was used for genetic map construction and conversion of recombination fraction into map distances in centiMorgans (cM) [41]. After preparing a framework genetic map, the remaining markers (distorted) were also integrated into the main framework map at recombination frequency (∂) of upto 50%. The final marker positions of each LG were then used to draw final genetic map using MapChart [42].

### Quantitative trait locus (QTL) analysis

Two genetic softwares were used for identification of main-effect QTLs (M-QTLs) (Windows QTLCartographer and QTLNetwork) and epistatic QTL interactions (E-QTLs) (QTLNetwork). Composite interval mapping (CIM) approach was used for identification of location and effect of M-QTLs using Windows QTLCartographer, version 2.5 [43] following the same criteria selected by Ravi et al. [26]. QTLCartographer uses a dynamic algorithm which considers various gene actions (additive and dominance), QTL-environment interactions and close linkage. Parameters such as model 6, scanning intervals of 1.0 cM between markers and putative QTLs with a window size of 10.0 cM were used for conducting the CIM analysis. In addition, forward-backward stepwise regression was selected for background control set by the number of marker cofactors along with 500 times permutations with 0.05 significance level and “Locate QTLs” option to locate QTLs.

Another software, QTLNetwork program ver. 2.0 [44] which is based on a mixed linear model, was used to identify M-QTLs and E-QTLs with the first-dimensional genome scan with the option to map epistasis and the second-dimensional genome scan to detect epistatic interactions with or without single-locus effect. Parameters such as 1000 permutations, experimental-wise significance level of 0.05 for detection of QTLs with their effect, genome scan configuration (1.0 cM walk speed, 10.0 cM testing window and filtration window size) and Monte Carlo Markov Chain (MCMC) for estimating QTL effects were selected for performing QTL analysis. QTL analysis was conducted on phenotyping data of individual year (trait_2010, trait_2011) for all the four traits namely oleic acid (C18:1), linoleic acid (C18:2), O/L ratio, and oil content.

## Additional files

Additional file 1:
**Genetic linkage map of the S-population.** This genetic map shows map location and order of all the 206 loci on the 20 linkage groups. Additional file 2:
**Genetic linkage map of the T-population.** This genetic map shows map location and order of all the 278 loci on the 20 linkage groups. Additional file 3:
**Genetic map order in different linkage groups of the S-population.**
Additional file 4:
**Genetic map order in different linkage groups of the T-population.**
Additional file 5:
**Summary of main-effect QTLs (M-QTLs) identified by QTLCartographer in the S-population.** This table shows details on location, flanking marker loci, LOD value, phenotypic variance explained and additive effects of 27 M-QTLs detected by QTLCartographer. Additional file 6:
**Summary of main-effect QTLs (M-QTLs) identified by QTLCartographer in the T-population.** This table shows details on location, flanking marker loci, LOD value, phenotypic variance explained and additive effects of 29 M-QTLs detected by QTLCartographer. Additional file 7:
**Summary of main-effect QTLs (M-QTLs) identified by QTLNetwork in the S-population.** This table shows details on location, flanking marker loci, phenotypic variance explained and additive effects of 11 M-QTLs detected by QTLNetwork. Additional file 8:
**Summary of main-effect QTLs (M-QTLs) identified by QTLNetwork in the T-population.** This table shows details on location, flanking marker loci, phenotypic variance explained and additive effects of 11 M-QTLs detected by QTLNetwork. Additional file 9:
**Summary of epistatatic QTLs (E-QTLs) identified by QTLNetwork in the S- and the T-populations.** This table shows details on search range, F value, phenotypic variance explained, number of interacting loci and names interacting loci for 10 E-QTLs detected by QTLNetwork. Additional file 10:
**Source of markers used for construction of genetic maps for the S- and the T-populations.** This table shows details on source of the markers used in this study.
