# Supplementary figures and images for: Identification of QTLs associated with oil content and mapping FAD2 genes and their relative contribution to oil quality in peanut (Arachis hypogaea L.)
Source: BMC Genet. 2014 Dec 10;15:133. doi: 10.1186/s12863-014-0133-4 (PMC4278341; doi:10.1186/s12863-014-0133-4)

## Slide 1
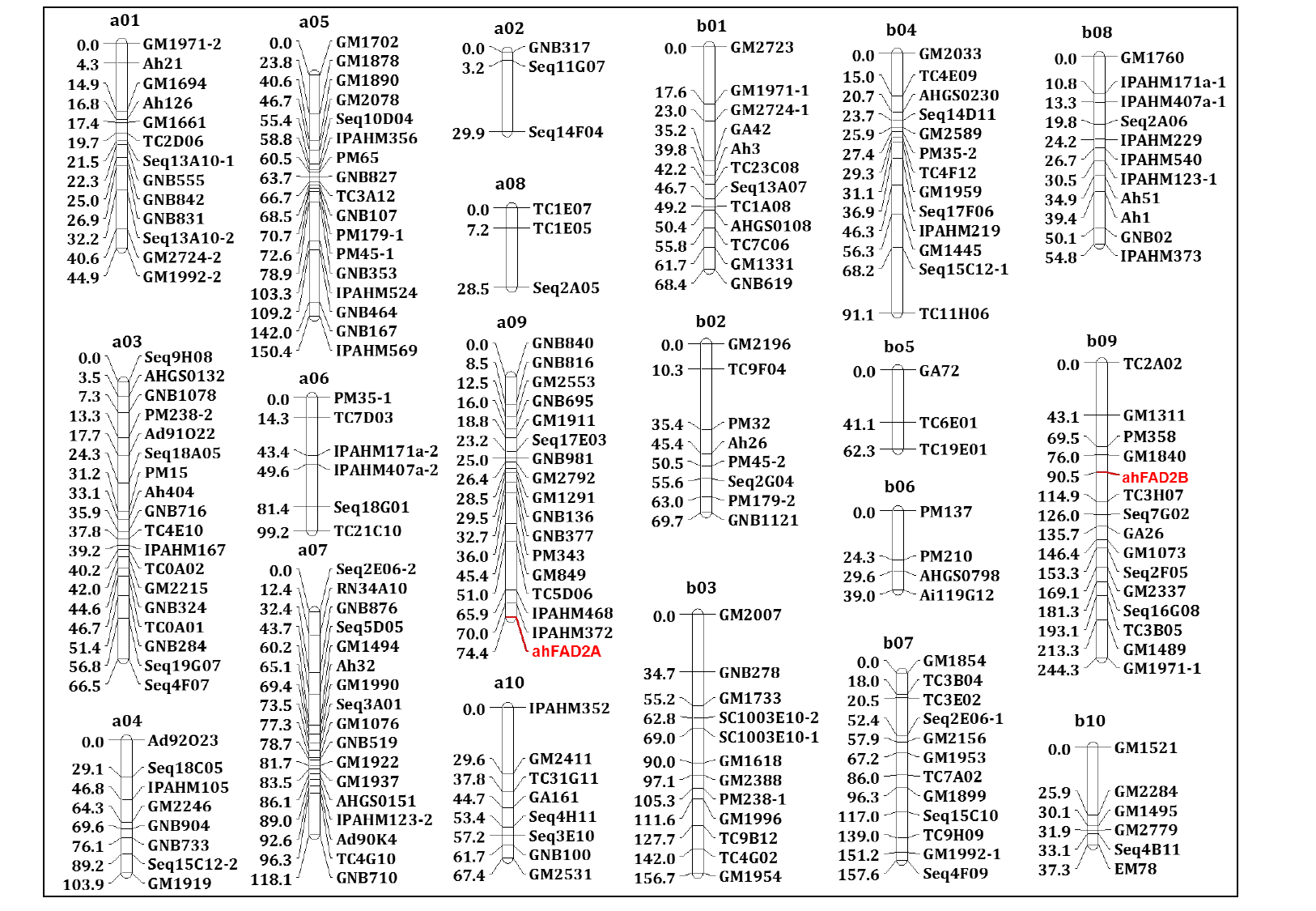

Supplement: Additional file 1: — Genetic linkage map of the S-population. This genetic map shows map location and order of all the 206 loci on the 20 linkage groups. [file 12863_2014_133_MOESM1_ESM.pptx]

## Slide 1
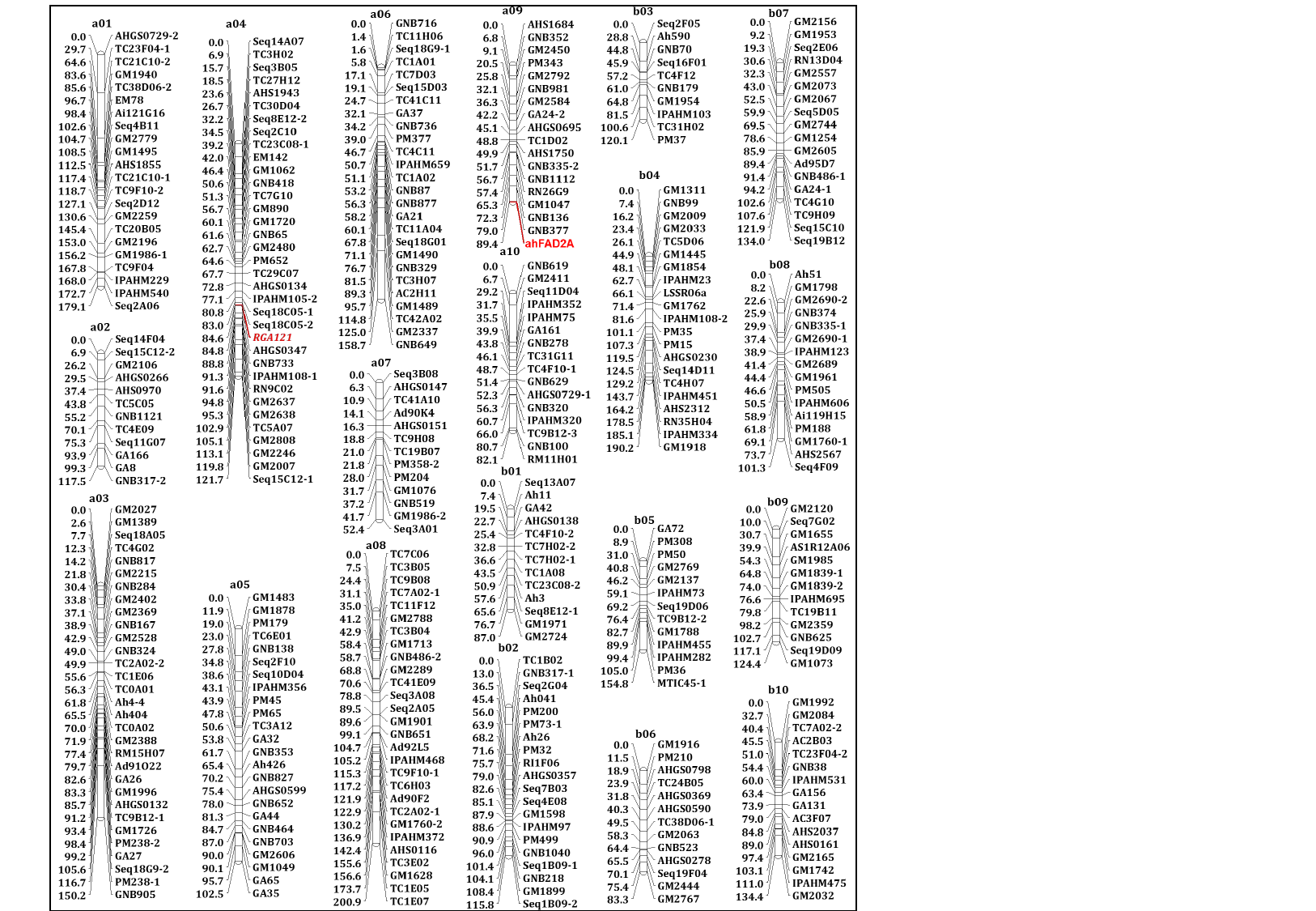

Supplement: Additional file 2: — Genetic linkage map of the T-population. This genetic map shows map location and order of all the 278 loci on the 20 linkage groups. [file 12863_2014_133_MOESM2_ESM.pptx]
